# The crystal structure of JNK from *Drosophila melanogaster* reveals an evolutionarily conserved topology with that of mammalian JNK proteins

**DOI:** 10.1186/s12900-015-0045-1

**Published:** 2015-09-16

**Authors:** Sarin Chimnaronk, Jatuporn Sitthiroongruang, Kanokporn Srisucharitpanit, Monrudee Srisaisup, Albert J. Ketterman, Panadda Boonserm

**Affiliations:** Institute of Molecular Biosciences, Mahidol University, Salaya, Phuttamonthon, Nakhon Pathom 73170 Thailand; Faculty of Allied Health Sciences, Burapha University, Mueang District, Saen Sook, Chonburi 20131 Thailand

## Abstract

**Background:**

The c-Jun N-terminal kinases (JNKs), members of the mitogen-activated protein kinase (MAPK) family, engage in diverse cellular responses to signals produced under normal development and stress conditions. In *Drosophila*, only one JNK member is present, whereas ten isoforms from three JNK genes (JNK1, 2, and 3) are present in mammalian cells. To date, several mammalian JNK structures have been determined, however, there has been no report of any insect JNK structure.

**Results:**

We report the first structure of JNK from *Drosophila melanogaster* (DJNK). The crystal structure of the unphosphorylated form of DJNK complexed with adenylyl imidodiphosphate (AMP-PNP) has been solved at 1.79 Å resolution. The fold and topology of DJNK are similar to those of mammalian JNK isoforms, demonstrating their evolutionarily conserved structures and functions. Structural comparisons of DJNK and the closely related mammalian JNKs also allow identification of putative catalytic residues, substrate-binding sites and conformational alterations upon docking interaction with *Drosophila* scaffold proteins.

**Conclusions:**

The DJNK structure reveals common features with those of the mammalian JNK isoforms, thereby allowing the mapping of putative catalytic and substrate binding sites. Additionally, structural changes upon peptide binding could be predicted based on the comparison with the closely-related JNK3 structure in complex with pepJIP1. This is the first structure of insect JNK reported to date, and will provide a platform for future mutational studies in *Drosophila* to ascertain the functional role of insect JNK.

**Electronic supplementary material:**

The online version of this article (doi:10.1186/s12900-015-0045-1) contains supplementary material, which is available to authorized users.

## Background

The c-Jun N-terminal kinases (JNKs), the extracellular signal-regulated kinases (ERK1/2) and the 38 kDa MAP kinases (p38 MAPKs) are members of the mitogen-activated protein kinase (MAPK) family [1]. Of the MAPK pathways, the JNK signaling pathway is of particular importance for various biological processes such as cell proliferation, differentiation, apoptosis, cellular responses to stress as well as embryonic development and morphogenesis [1]. Activation of JNKs requires a dual phosphorylation, by upstream JNK kinases (MKK4 and MKK7), on two conserved threonine and tyrosine residues within the Thr-X-Tyr motif in their activation loops [2]. The phosphorylated JNKs subsequently activate their substrates such as transcriptional factors, resulting in mediating the gene expression regulation in response to stress stimuli.

In mammalian cells, at least 10 different splicing isoforms of three JNK genes (JNK1, 2, and 3) have been identified [2]. JNK1 and JNK2 are widely expressed in a variety of tissues, whereas JNK3 is selectively expressed in the brain, heart and testis [2]. Similar to mammalian cells, the JNK signaling pathway is also conserved in *Drosophila*. The *Drosophila* JNK pathway consists of *Drosophila* JNK or basket (DJNK) and JNK kinase Hep, which are homologs of JNK and MKK7 in mammals, respectively [3]. The important role of *Drosophila* JNK pathway in embryogenesis has been emphasized by the findings that mutations in the *Drosophila* JNK pathway components resulted in the disruption of the morphogenetic process of dorsal closure during embryogenesis [4]. Although there are ten JNK isoforms in mammals, only one JNK isoform (DJNK) exists in *Drosophila* [4]. Due to a lower genetic complexity and redundancy of the JNK signaling components in comparison to their mammalian counterparts, the *Drosophila* JNK pathway thus becomes a simpler model system for a study of JNK regulation under normal development and stress conditions.

Several efforts have been made to determine the structures of different mammalian JNK isoforms either in the presence or absence of the JNK pathway scaffolding proteins. In contrast, the structural information of DJNK and other related *Drosophila* JNK signaling components is still unavailable. In this study, we have solved the crystal structure of unphosphorylated DJNK in complex with adenylyl imidodiphosphate (AMP-PNP) and magnesium at 1.79 Å resolution. This is the first insect JNK structure to be solved and, together with the mammalian JNK structures, provides crucial insights into the evolutionary conservation of structures and catalytic regulation across insect and mammalian JNK proteins.

## Methods

### Protein expression and purification

The *Drosophila* JNK gene (*DJNK* or *basket* [GenBank: AAB97094.1]) was cloned in-frame with the hexahistidine tag at the N-terminus as previously described [5]. *E. coli* BL21 (DE3)pLysS cells containing the recombinant plasmid were grown in Luria-Bertani (LB) broth containing 100 μg/ml kanamycin and 34 μg/ml chloramphenicol at 37 °C until an OD_600_ reached 0.6. The protein expression was induced by the addition of IPTG (isopropyl β-D thiogalactopyranoside) at a final concentration of 0.2 mM. The cultured cells were further grown for 15 h at 25 °C, and harvested by centrifugation at 6000 × g for 10 min. Cell pellet was resuspended in 20 ml of buffer A (20 mM sodium phosphate buffer pH 7.4, 0.5 M NaCl) containing 4 mg/ml lysozyme and 1 mM β-mercaptoethanol. Cells were disrupted by the French press at 1000 psi, and the lysate was clarified by centrifugation at 10,000 × g for 30 min, followed by being filtered through a 0.22 μm membrane. The supernatant was loaded onto a 5-ml HiTrap™ Chelating HP column packed with precharged Ni^2+^ resin (GE Healthcare Life Sciences). The bound (His)_6_-tagged JNK protein was eluted by buffer A containing 200 mM imidazole. The imidazole was further removed using a HiTrap^TM^ desalting column (GE Healthcare Life Sciences) pre-equilibrated with 50 mM Tris–HCl, pH 8.0 and 10 mM DTT. The eluted fractions were concentrated to 10 mg/ml by using a Microsep-10 (PALL). The proteins collected at every step were analyzed by 12 % sodium dodecyl sulfate–polyacrylamide electrophoresis (SDS-PAGE). The concentration of protein was determined by Bradford’s assay using BSA as standard protein.

### Protein crystallization

Crystallization trials were conducted using hanging- and sitting-drop vapour-diffusion methods in 24 and 96-well plates at 295 K (Molecular Dimensions, UK and QIAGEN, Germany). Before setting up the crystallization, the purified DJNK was mixed with a substrate, AMP-PNP (Adenosine 5′-(β,γ-imido) triphosphate lithium salt hydrate) and MgCl_2_ at the concentrations of 1 mM and 2 mM, respectively. Initial screening was performed using Hampton Research Crystal Screen kits (Hampton Research, USA) and positive hits were then optimized. Drops were prepared by mixing 1.2 μl of 10 mg/ml protein with an equivalent volume of reservoir solution and were equilibrated against 500 μl of reservoir solution. After optimizing the conditions, prism crystals were obtained from a condition consisting of 25 % (w/v) PEG 4000, 0.1 M Tris–HCl pH 8.5 and 0.2 M MgCl_2_. The crystals grew to a maximum size of about 50 × 100 μm within 1 month after incubation at 20 °C.

### X-ray data collection and structural determination

Preliminary X-ray diffraction was performed using an in-house X-ray source (MICROSTAR^TM^, BRUKER, Thailand) at the MX end station at Synchrotron Light Research Institute (SLRI, Thailand), whereas high resolution data were collected at the beamline BL32XU of the SPring-8 synchrotron (Hyogo, Japan). Prior to data collection, a single crystal was briefly soaked in its reservoir solution containing 12 % sucrose as a cryoprotectant before being flash-frozen in the nitrogen stream at 100 K. A total of 560 images were collected with an oscillation angle of 0.2° at a crystal-to-detector distance of 150 mm. The exposure time per image was set to 1 s. The X-ray diffraction pattern images were processed and scaled using XDS program package [6]. The structure was solved with the molecular replacement program MOLREP in *CCP*4 program suite [7] using the crystal structure of human JNK1 [PDB:2XS0 [8]] as a search model. The electron density map was calculated and a model was built with the graphic software COOT [9]. Several rounds of refinement were performed using REFMAC5 [10]. Ligand and water molecules were added at nearly the final step of refinement and manually validated. The final model including water molecules was justified by monitoring the *R*_factor_ and *R*_free_. The quality of protein structure was determined by MolProbity [11]. Statistics of data collection and refinement are summarized in Table 1. The residues falling outside the allowed region were modified by adjusting torsion angles, Phi (Φ), Psi (ψ). All images of the DJNK structure were prepared with PyMOL (Delano Scientific, Palo Alto, CA, http://www.pymol.org/).

**Table 1:** Data collection and refinement statistics of DJNK structure

Data collection	
Space group	*P*2_1_2_1_2_1_
Unit-cell parameters (Å)	*a* = 52.49, *b* = 55.33, *c* = 126.72
High resolution limit (Å)	1.58
Total reflections	219226
Unique reflections	51133
Average mosaicity (°)	0.0
Completeness (%)	99.4 (97.6)^a^
Redundancy	4.3(4.2)^a^
^ b^ *R*_merge_ (%)	6.2 (79.4)^a^
* I/*σ*(I)*	11.8 (1.8)^a^
Refinement statistics	
Resolution range (Å)	28.28-1.79
Highest resolution shell (Å)	1.84-1.79
^ c^ *R*-factor (%)	18.9
^ d^ *R*_free_-factor (%)	22.4
Averaged B-factor (Å^2^)	26.0
Number of non-hydrogen atoms	
Protein	2810
Water	143
ADPNP	1
Mg^2+^ ions	2
RMSD from ideal geometry	
Bond length (Å)	1.07
Bond angle (°)	1.06
^ e^Ramachandran plot (%)	
Favored regions	97.0
Additional allowed regions	3.0
Outliers	0

^a^Number in parentheses refer to the outer resolution shell

^b^ *R*_merge_ =  ∑_*hkl*_∑_*i*_|*I*_*hkl,i*_ – 〈*I*_*hkl*_〉|/ ∑_*hkl*_∑_*i*_ *I*_*hkl,i*_, where 〈*I*_*hkl*_〉 is the mean intensity of symmetry-equivalent reflection

^c^ *R*-factor = ∑ |*F*_obs_-*F*_cal_|/∑*F*_obs_, where *F*_obs_ and *F*_cal_ are observed and calculated structure factor amplitudes, respectively

^d^ *R*_free_-factor value was calculated as *R*-factor but using a subset (10 %) of reflections that were not used for refinement

^e^Ramachandran plot was calculated using MolProbity [11]

### Accession number

Atomic coordinates and structure factor amplitudes of DJNK have been deposited into the Protein Data Bank (PDB) with the accession code 5AWM.

## Results and discussion

### Overall structure of DJNK

**Fig. 1:**
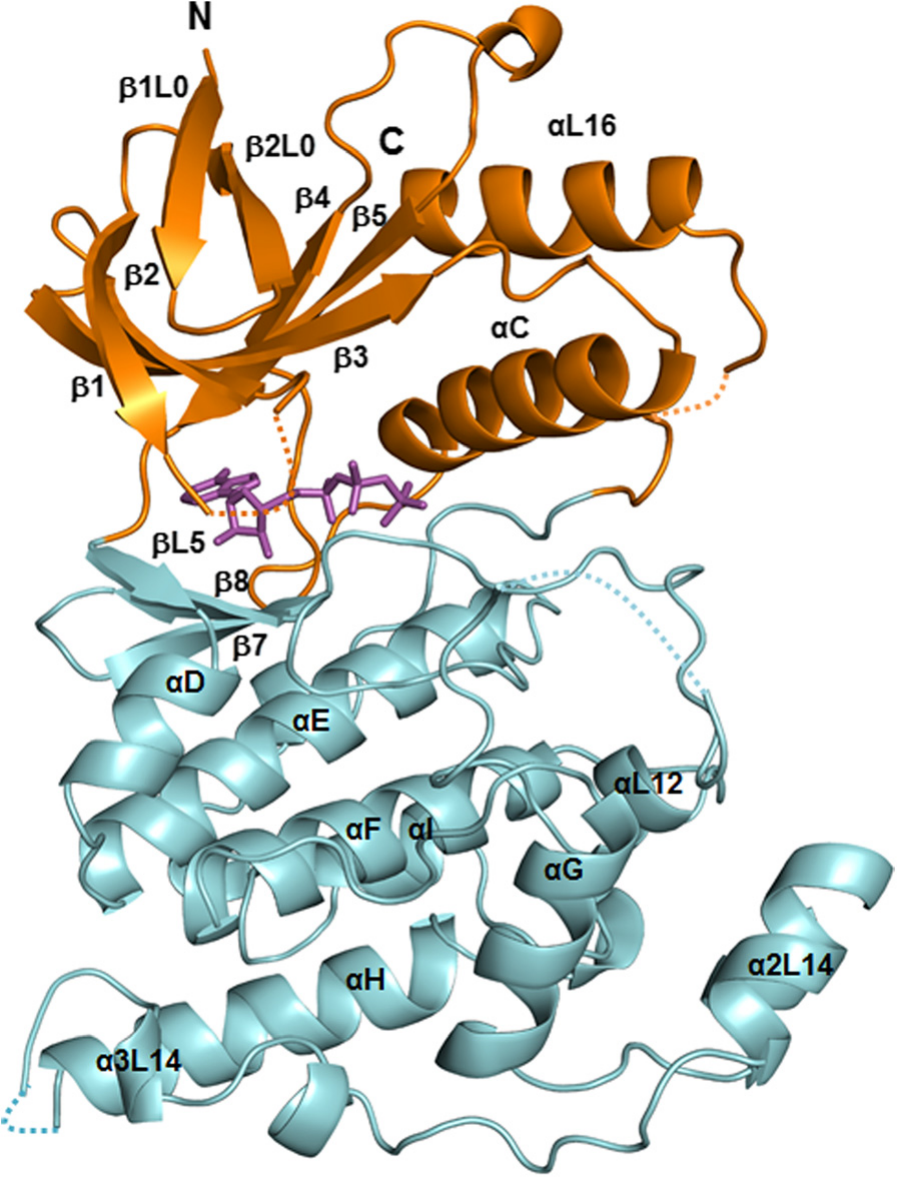
Ribbon representation of *Drosophila* JNK complexed with AMP-PNP at a resolution of 1.79 Å. The DJNK structure is formed by two distinct domains: the N-terminal domain (orange) and the C-terminal domain (cyan). The bound AMP-PNP located in a deep cleft at the domain interface is shown in a magenta stick model. Secondary-structure elements are labeled as described in additional file 1. The disordered connecting loops are shown by dotted lines

The crystal structure of *Drosophila* JNK (DJNK) has been solved to 1.79 Å resolution and consists of 354 amino acid residues (Gln7-Tyr362) with one chemical compound of AMP-PNP (ATP analog) bound at the active site cleft (Fig. 1). The electron densities are not visible in four loop regions encompassing residues Ser32-Gln35, Thr181-Tyr183, Asn282-Asn283, Asp342-Glu343, likely due to structural disorder. The overall DJNK structure is composed of two distinct domains. The N-terminal domain (residues 7–109 and residues 338–362) consists of seven β strands (β1L0, β2L0, β1, β2, β3, β4, and β5) and two α helices (αC and αL16), while the C-terminal domain (residues 110–172 and residues 187–337) contains mostly α helices (αD, αE, αF, αG, αH, αI, αL12, α1L14, α2L14, α3L14 and αIL16,) with five short 3_10_-helices and three β strands (βL5, β7, and β8) (Fig. 1). The two domains are connected by the so-called hinge regions (residues 107–111 and residues 330–348). A deep cleft at the domain interface contains the ATP-binding site (Fig. 1). DJNK also possesses extensions and insertions characteristic for MAP kinases including an N-terminal β-hairpin (β1L0 and β2L0), an extended loop between αG and α1L14, α1L14, α2L14, α3L14, and αL16.

Structural comparisons of DJNK with other known JNK structures was performed using the DALI server [12]. As expected, the overall structure of DJNK shows a similar protein kinase fold to that of mammalian JNK1 [PDB:1UKH [13]], mammalian JNK2 [PDB:3E7O [14]] and mammalian JNK3 [PDB:1JNK [15]] with amino acid sequence identities of 82, 77 and 79 %, respectively (see Additional file 1).

### The highly-flexible activation loop

The activation loop of DJNK, also referred to as the phosphorylation lip, spans residues Leu166 to Ala191. This region contains the dual phosphorylation sites phosphorylated by specific upstream MAP kinase kinases. These residues, Thr181 and Tyr183, are found in the conserved Thr-X-Tyr motif. Despite the common regulatory mechanism of dual phosphorylation, the number of residues in the activation loops among protein kinase family members is variable [16]. No electron density, however, is visible for residues Thr181-Tyr183, including the two phosphorylation sites in the activation loop of DJNK, implying its high flexibility in the unphosphorylated form. The similar disordered phosphorylation sites are also found in the unphosphorylated forms of JNK2 and JNK3 [14, 15, 17]. On the other hand, one of the two protein chains of JNK2 reveals a well-ordered activation loop which is mainly stabilized by crystal packing interactions. Based on these observations, it could be assumed that both Thr181 and Tyr183 of DJNK are solvent-exposed and do not participate in crystal packing interaction similarly to the corresponding residues Thr221 and Tyr223 in the unphosphorylated JNK3 [15], thereby becoming readily accessible to the JNK upstream activating kinases. Although the activation loop of DJNK is assumed to adopt multiple conformations in solution, the restructuring of this loop to adopt an appropriate conformation upon phosphorylation to allow the enzyme to recognize the specific substrate may also occur. Hence, the inherent flexibility of DJNK activation loop may play a central role in the enzymatic regulation upon substrate binding.

### The putative conformational changes of the catalytic site upon peptide binding

**Fig. 2:**
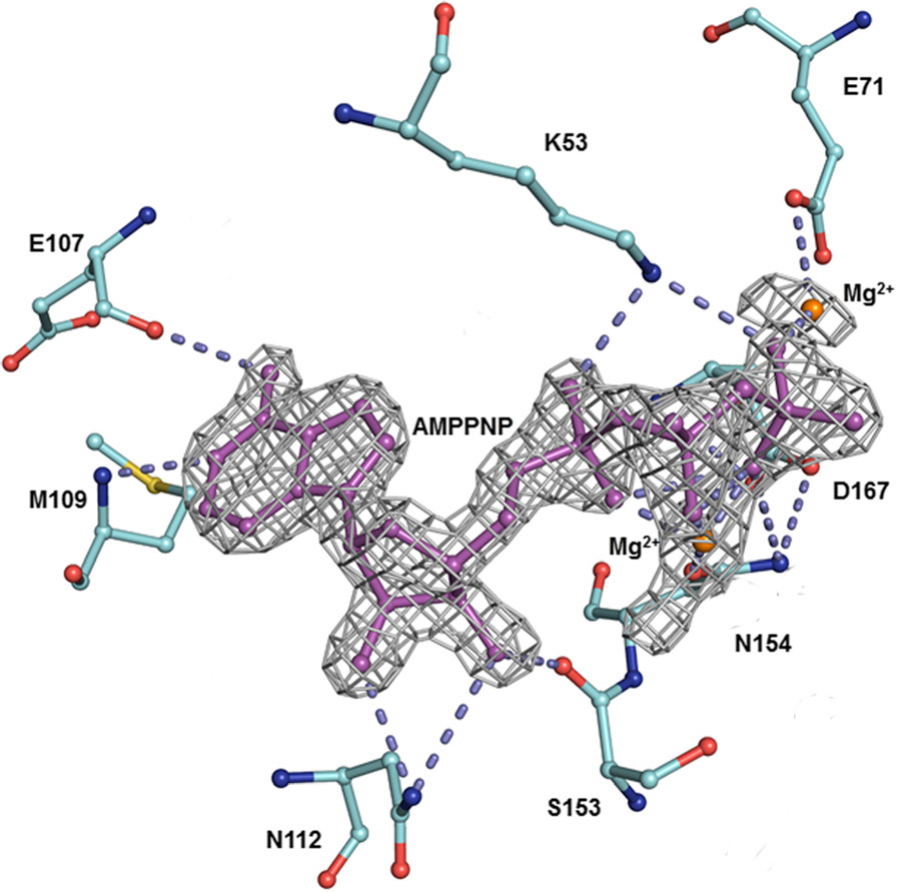
AMP-PNP bound to *Drosophila* JNK. *F*_o_-*F*_c_ omit electron density map (contoured at 4σ) is shown in grey for the bound DJNK-AMP-PNP (magenta stick model). Amino acid residues crucial for the ATP binding of DJNK are shown in cyan. Two Mg^2+^ ions are shown as orange balls. Hydrogen bonds are indicated as dashed lines

In the structure of DJNK complexed with AMP-PNP, the AMP-PNP is bound in a deep cleft between the N- and C-terminal domains. The glycine-rich sequence (Gly31-Ser–Gly-Ala-Gln-Gly-Ile-Val38) of DJNK, however, is not well defined, implicating that this loop is highly flexible and does not directly interact with AMP-PNP. The residues important for the ATP binding come from different parts of the DJNK structure. The orientation of AMP-PNP in DJNK is determined by the hydrogen bonds between the phosphate groups of AMP-PNP and the backbone amide of Met109 and backbone carbonyl groups of Glu107 and Ser153. The side chain of Lys53, a putative catalytic residue which is highly conserved within the MAP kinase family [15], is directly involved in the formation of hydrogen bonds with the α- and β-phosphoryl groups of the nucleotide, thereby playing a key role in the proper positioning of ATP in MAP kinases. Glu107 and Asn112, found to be conserved among JNK isoforms, also make hydrogen bonds with the phosphate groups. Two fixed magnesium ions are observed in the DJNK. The side chain carbonyl groups of Asn154 and Glu71 appear to interact with the phosphate groups of AMP-PNP through the Mg^2+^ ions. An important role in metal chelation has been proposed for Asp184 in cAMP-dependent protein kinase (cAPK), corresponding to Asp167 of DJNK, which forms direct interactions with the metal ions [18]. The side chain of Asp167 of DJNK does not directly interact with the metal ion, however, it is connected to the metal ion through an interaction with the side chain of Asn154. A similar interaction is also observed between Asp207 and Asn194 of JNK3 (Fig. 2) [15].

**Fig. 3:**
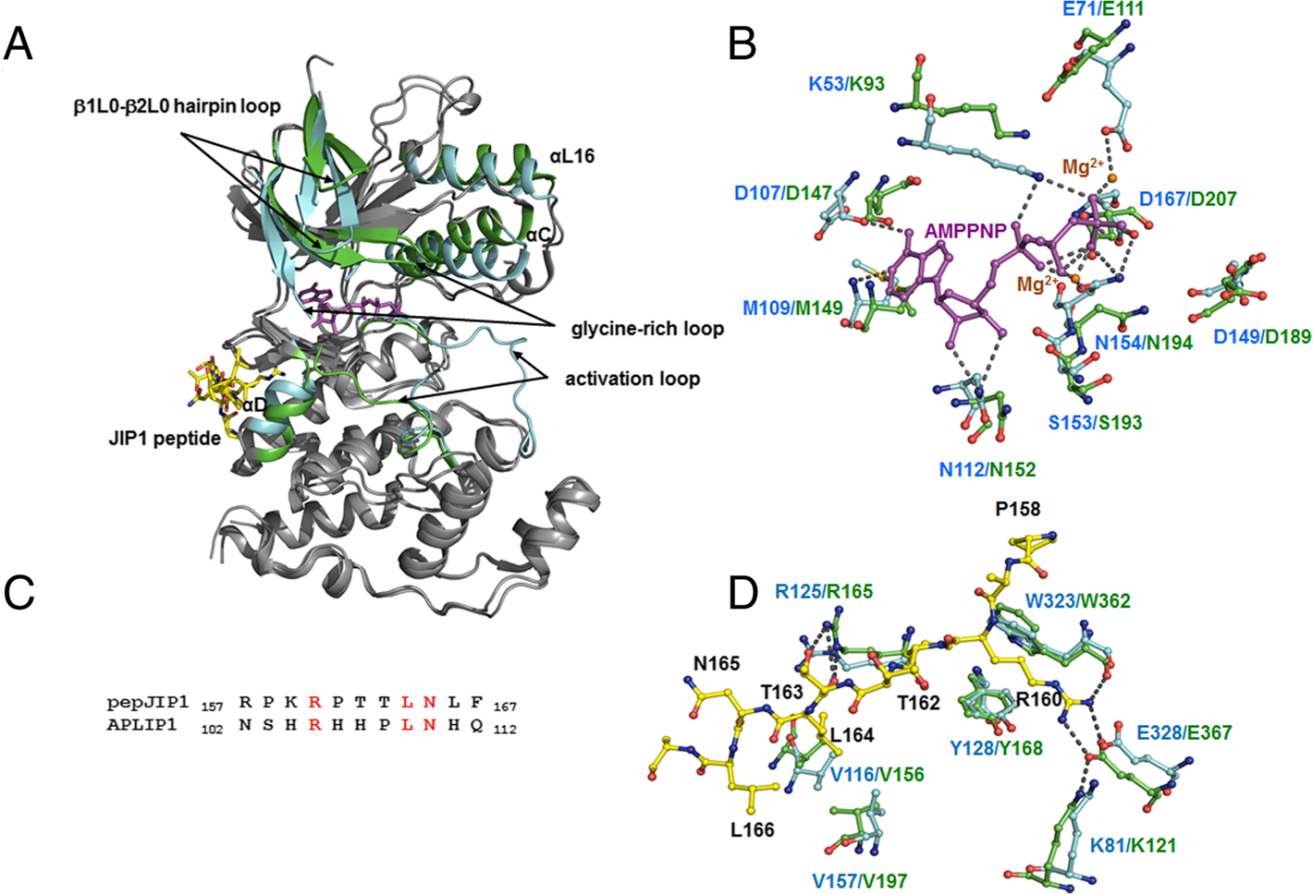
The putative conformational changes of DJNK upon peptide binding. **a** Global superposition of the DJNK bound to AMP-PNP with the JNK3-pepJIP1 complex. The regions of major structural changes upon peptide binding including the glycine-rich loop, the activation loop, the N-terminal MAPK insert (β1L0-β2L0 hairpin), αL16, αC, and αD are highlighted in green for JNK3-pJIP1 and cyan for DJNK, respectively, whereas the homologous regions are in grey color. **b** Close-up view of conformational changes in the ATP-binding and catalytic sites. Amino acid residues crucial for the ATP binding and catalytic activity of DJNK and JNK3-pepJIP1 are shown in cyan and green, respectively. The bound DJNK-AMP-PNP is shown in a magenta stick model. Two Mg^2+^ ions are shown as orange balls. Hydrogen bonds are indicated as dashed lines. **c** Sequence alignment of the docking sites of the scaffolding proteins JIP1 and APLIP1. The conserved residues are highlighted in red. **d** Close-up view of superposition of DJNK and JNK3-pepJIP1 in the peptide-binding sites. Amino acid residues crucial for the scaffold protein binding of DJNK and JNK3-pepJIP1 are labeled as in panel **b**, whereas the residues of pepJIP1 are presented in yellow sticks and labeled in black

To gain insights into the conformational changes upon peptide binding, the structure of JNK3 complexed with 11-mer JIP1 peptide (pepJIP1), a peptide of JNK scaffolding protein, was compared with the structure of peptide-free DJNK. In JNK3-pepJIP1 structure [PDB:4H39 [17]], part of the activation loop coils into a helix that partially occupies the ATP binding site. When the full structures of DJNK and JNK3-pepJIP1 complex were superimposed, the root-mean-square (r.m.s.) Cα distance was increased by ~1 Å compared with that from the superposition with peptide-free JNK3. Major structural changes upon peptide binding were observed in several regions especially in the N-terminal domain. In particular, the β1-β2 and glycine-rich loop of the JNK3-pepJIP1 was shifted by ~6 Å relative to the corresponding regions of the DJNK (Fig 3a). This substantial shift is required to allow space for the activation loop to dock into the ATP binding site of JNK3 (Fig. 3a). As a result of the shift in glycine-rich loop, the β1L0-β2L0 hairpin loop of JNK3-pepJIP1 is moved away by ~8 Å relative to the corresponding loop of DJNK to avoid the steric clashes.

The conserved aspartic acids in both HRDLKXXN and DFG motifs in the C-terminal domain, corresponding to Asp149 and Asp167 of DJNK, have been thought to be essential for protein kinase activity as previously described [19]. These two catalytic residues together with two other conserved key residues, Lys53 and Glu71 in the N-terminal domain of DJNK, are compared with the corresponding residues of JNK3-pepJIP1. Most of the catalytic residues of DJNK are in slightly different positions compared to their corresponding residues in JNK3-pepJIP1 (Fig. 3b). The disruption of hydrogen bond network formed between Asn194, Glu207 and Mg^2+^ ions, known as a requisite for catalysis, is apparent in JNK3-pepJIP1 structure (Fig. 3b). The peptide-induced conformational changes also involve a shift in αC helix, consequently disrupting the hydrogen bonding interactions of residues in the αC helix with those in the activation loop (Fig. 3a). The shifted αC is also associated with a subtle move of the N-terminal MAPK insert αL16 (Fig. 3a).

Taken together, the conformational changes upon pepJIP1 binding potentially blocks the formation of active catalytic sites as a result of the failure to properly bind to ATP. This allosteric inhibition mechanism, nonetheless, is likely reversible once the activation loop is phophorylated, allowing the catalytically active conformation to form. Considering that the ATP binding sites and catalytically important residues are evolutionarily conserved among mammalian JNK isoforms and DJNK, the different JNKs thus likely undergo common structural transformations during peptide binding.

### The putative peptide-binding and docking sites

In the MAPK signaling pathway, docking interactions are formed between the specific conserved regions on MAPKs, so called common docking groove or CD groove, and their interacting molecules. Specificity and enzymatic reactions are determined by the specific interactions of CD grooves with specific docking sites or docking motifs commonly found in transcription factors, upstream activating kinases, phosphatases, scaffold proteins, and substrates [20]. The docking sites possess both basic and hydrophobic sequences in the arrangement of (R/K)_2–3_-(X)_1–6_-Ø_A_-X-Ø_B_ (where Ø_A_ and Ø_B_ are hydrophobic residues) [20]. Despite sharing common linear motifs, variations in sequences of CD grooves and docking sites appear to be an important determinant of pathway specificity for the different MAPKs.

JNK-interacting protein-1 (JIP1) was identified as a scaffold protein of the JNK module. The mammalian JIP homolog, namely APLIP1 (β-Amyloid Precursor-Like Interacting Protein 1), was identified in *Drosophila melanogaster* [21]. The similarities of APLIP1 to the mammalian homologs JIP1 and JIP2 include abundant expression in neural tissues, interactions with component(s) of the JNK signaling pathway and with the motor kinesin, and formation of homo-oligomers [21], reflecting their conserved functions and structures. Based on amino acid sequence alignment of mammalian JIP1 and *Drosophila* APLIP1 (Accession numbers Q9UQF2 and Q9W0K0, respectively), a putative docking motif could be mapped at positions 102–112 of APLIP1 (Fig. 3c, also see Additional file 2). Thus, although the structure of DJNK has been solved in the unbound state, it is still possible to understand some aspects of the docking specificity via both primary sequence and three-dimensional structure comparisons.

Superposition of DJNK and JNK3-pepJIP1 reveals that amino acid residues in their CD grooves, corresponding to those on the C-terminal domain surface covering αD, αE, and β7-β8 reverse turn, are conserved and homologous in positions (Fig. 3d). The side chain of Arg160 of pepJIP1, which is at equivalent position to Arg105 of APLIP1, rotates to form an extensive electrostatic network with Glu367 (in αIL16) and Lys121 (in loop following αC) of JNK3. These residues are well superimposed on their corresponding DJNK Glu328 and Lys81 residues, implying the presence of a conserved electrostatic network in DJNK (Fig. 3d). The pepJIP1 Arg160 also forms a hydrogen bond with the backbone carbonyl oxygen atom of JNK3 Trp362, conserved in position with Trp323 of DJNK. In fact, the critical importance of Arg160 in pepJIP1 was verified by ITC measurement as substitutions of this residue conferred significant lower affinity of JNK3-pepJIP1 binding [17]. Another conserved pepJIP1 Leu164 residue (Ø_A_ in the Ø_A_-X-Ø_B_ motif), corresponding to Leu109 of APLIP1, makes van der Waal contacts with Val156 and Val197 of JNK3 which are also conserved in positions with Val116 and Val157 of DJNK, respectively. These two conserved basic and hydrophobic residues in the docking sites of JIP1 and APLIP1 thus serve as anchor points for interacting with the common surface features of JNKs. On the other hand, pepJIP1 Asn165, conserved with APLIP1 Asn110, does not have any interaction with JNK3 but it forms a hydrogen bond with the backbone nitrogen atom of pepJIP1 Phe167, suggesting a role for this conserved residue in local folding of the peptide.

Despite the presence of consensus residues in the docking site, APLIP1 failed to interact with DJNK under experimental conditions *in vitro* [21]. This seems to be associated with the lower affinity of DJNK and APLIP1 binding, compared with the JNK3-pepJIP1 counterpart. A notable difference is that one of the key hydrophobic residues corresponding to Ø_B_ in the Ø_A_-X-Ø_B_ motif is replaced by His111 in APLIP1, thus likely contributing to the weak hydrophobic interactions with the hydrophobic pocket of DJNK docking groove. In conjunction with the hydrophobic motif, the variable amino acids located between the conserved anchor points also could be important determinants of the ineffective docking interaction of DJNK and APLIP1. On the other hand, APLIP1 was found to bind to *Drosophila* Hep, a protein kinase upstream of DJNK, which is functionally similar to mammalian JIP1 [21]. Thus, APLIP1 shares most of the features in common with mammalian JIP1, except for the binding to JNK. Therefore, a structural basis of DJNK binding specificity with its binding partners remains to be elucidated via biochemical assays and co-crystallization with the peptide.

## Conclusions

This is the first structural determination of *Drosophila* JNK, thereby serving as a representative of insect JNKs. The structure reveals common architectures with those of the mammalian JNK isoforms, allowing the identification of putative catalytic and substrate binding sites. Structural changes induced by peptide binding could be anticipated based on the comparison with the closely-related JNK3 structure in complex with pepJIP1. Although the evidence of specificity of DJNK with partner proteins is still limited, the structure and sequence alignments provide some clues for the docking interaction of DJNK with its putative scaffold protein.

### Availability of supporting data

The data sets supporting the results of this article are available in the Protein Data Bank Japan (PDBj), Accession Code 5AWM in http://pdbj.org/mine/summary/5awm.

## Additional files

Additional file 1:
**Structure-based sequence alignment of DJNK, mammalian JNK1, JNK2, and JNK3.** (DOCX 46 kb) Additional file 2:
**Pairwise sequence alignment of mammalian JIP1 and** ***Drosophila melanogaster*** **APLIP1 [UniProt:Q9UQF2 and UniProt:Q9W0K0, respectively].** (DOCX 24 kb)

## Abbreviations

JNK: c-Jun N-terminal kinase
DJNK: *Drosophila* JNK
