# Supplementary material for: The crystal structure of JNK from Drosophila melanogaster reveals an evolutionarily conserved topology with that of mammalian JNK proteins
Source: BMC Struct Biol. 2015 Sep 16;15:17. doi: 10.1186/s12900-015-0045-1 (PMC4573485; doi:10.1186/s12900-015-0045-1)
Supplement: Additional file 1: — Structure-based sequence alignment of DJNK, mammalian JNK1, JNK2, and JNK3. (DOCX 46 kb) [file 12900_2015_45_MOESM1_ESM.docx]

**The Crystal Structure of JNK from *Drosophila melanogaster* Reveals an Evolutionarily Conserved Topology with that of Mammalian JNK Proteins.**

Sarin Chimnaronk^1^ , Jatuporn Sitthiroongruang^1^, Kanokporn Srisucharitpanit^2^, Monrudee Srisaisup^1^, Albert J. Ketterman^1^, Panadda Boonserm^1*^

^1^ Institute of Molecular Biosciences, Mahidol University, Salaya, Phuttamonthon, Nakhon Pathom 73170, Thailand

^2^ Faculty of Allied Health Sciences, Burapha University, Saen Sook, Mueang District, Chonburi 20131, Thailand

E-mails: Sarin Chimnaronk - sarin.chim@mahidol.ac.th; Jatuporn Sitthiroongruang - robben16_chelsea@hotmail.com; Kanokporn Srisucharitpanit - kanokporns@buu.ac.th; Monrudee Srisaisup – catta_w255@hotmail.co.th; Albert J. Ketterman - albert.ket@mahidol.ac.th; Panadda Boonserm* - panadda.boo@mahidol.ac.th

*Corresponding author

DJNK QHYTVEVGDTNFTIHSRYINLRPIG*SGAQ*GIVCAAYDTITQQNVAIKKLSRPFQNVTHAK 66

JNK1 NFYSVEIGDSTFTVLKRYQNLKPIGSGAQGIVCAAYDAILERNVAIKKLSRPFQNQTHAK 68

JNK2 QFYSVQVADSTFTVLKRYQQLKPIGSGAQGIVCAAFDTVLGINVAVKKLSRPFQNQTHAK 68

JNK3 QFYSVEVGDSTFTVLKRYQNLKPIGSGAQGIVCAAYDAVLDRNVAIKKLSRPFQNQTHAK 106

DJNK RAYREFKLMKLVNHKNIIGLLNAFTPQRNLEEFQDVYLVMELMDANLCQVIQMDLDHDRM 126

JNK1 RAYRELVLMKCVNHKNIIGLLNVFTPQKSLEEFQDVYIVMELMDANLCQVIQMELDHERM 128

JNK2 RAYRELVLLKCVNHKNIISLLNVFTPQKTLEEFQDVYLVMELMDANLCQVIHMELDHERM 128

JNK3 RAYRELVLMKCVNHKNIISLLNVFTPQKTLEEFQDVYLVMELMDANLCQVIQMELDHERM 166


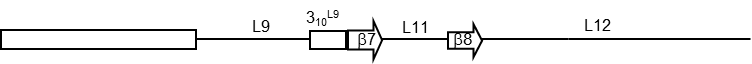


DJNK SYLLYQMLCGIKHLHSAGIIHRDLKPSNIVVKADCTLKILDFGLARTAGTTFMM*TPY*VVT 186

JNK1 SYLLYQMLCGIKHLHSAGIIHRDLKPSNIVVKSDCTLKILDFGL*ARTAGTSFMMTPYVVT* 188

JNK2 SYLLYQMLCGIKHLHSAGIIHRDLKPSNIVVKSDCTLKILDFGLARTASTNFMMTPYVVT 188

JNK3 SYLLYQMLCGIKHLHSAGIIHRDLKPSNIVVKSDCTLKILDFGLA*RTAGT*SFMMTPYVVT 226

* *

DJNK RYYRAPEVILGMGYTENVDIWSVGCIMGEMIRGGVLFPGTDHIDQWNKIIEQLGTPSPSF 246

JNK1 *R*YYRAPEVILGMGYKENVDIWSVGCIMGEMIKGGVLFPGTDHIDQWNKVIEQLGTPCPEF 248

JNK2 RYYRAPEVILGMGYKENVDIWSVGCIMGELVKGSVIFQGTDHIDQWNKVIEQLGTPSAEF 248

JNK3 RYYRAPEVILGMGYKENVDIWSVGCIMGEMVRHKILFPGRDYIDQWNKVIEQLGTPCPEF 286


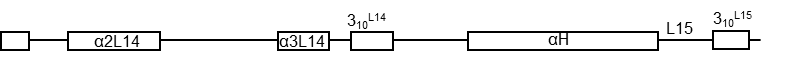


DJNK MQRLQPTVRNYVENRPRYTGYSFDRLFPDGLFPND*NN*QNSRRKASDARNLLSKMLVIDPEQ 307

JNK1 MKKLQPTVRTYVENRPKYAGYSFEKLFPDVLFP*ADSEH*N-KLKASQARDLLSKMLVIDASK 308

JNK2 MAALQPTVRNYVENRPAYPGIAFEELFPDWIFP*SE*SERD-KIKTSQARDLLSKMLVIDPDK 308

JNK3 MKKLQPTVRNYVENRPKYAGLTFPKLFPDSLFPADSEHN-KLKASQARDLLSKMLVIDPAK 346


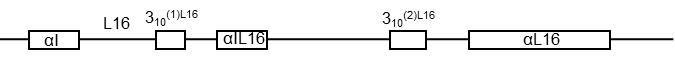


DJNK RISVDEALKHEYINVWYDAEEVDAPAPEPYDHSV*DE*REHTVEQWKELIYEEVMDY 362

JNK1 RISVDEALQHPYINVWYDPSEAEAPPPK*IPDKQLDEREHT*IEEWKELIYKEVMDL 361

JNK2 RISVDEALRHPYITVWYDPAEAEAPPPQI*YDAQLEERE*HAIEEWKELIYKEVM 361

JNK3 RISVDDALQHPYINVWYDPAEVEAPPP*QIYDK*QLDEREHTIEEWKELIYKEVMN 400

**Additional file 1.** Structure-based sequence alignment of DJNK, mammalian JNK1, JNK2, and JNK3. The amino acid sequences of DJNK, JNK1 [PDB:1UKH ^13^], JNK2 [PDB:3E7O ^14^] and JNK3 [PDB:1JNK ^15^] are aligned based on structural comparisons. The secondary structure elements for DJNK are indicated above the sequences with open boxes designating *α* and 3_10_ helices and open arrows designating *β* strands. Residues in *italic letters* are not included in the models. Phosphorylation sites are denoted by asterisks. Conserved amino acids crucial for the ATP binding, catalytic activity and peptide binding are highlighted.
