# Supplementary material for: The crystal structure of JNK from Drosophila melanogaster reveals an evolutionarily conserved topology with that of mammalian JNK proteins
Source: BMC Struct Biol. 2015 Sep 16;15:17. doi: 10.1186/s12900-015-0045-1 (PMC4573485; doi:10.1186/s12900-015-0045-1)
Supplement: Additional file 2: — Pairwise sequence alignment of mammalian JIP1 and Drosophila melanogaster APLIP1 [UniProt:Q9UQF2 and UniProt:Q9W0K0, respectively]. (DOCX 24 kb) [file 12900_2015_45_MOESM2_ESM.docx]

**The Crystal Structure of JNK from *Drosophila melanogaster* Reveals an Evolutionarily Conserved Topology with that of Mammalian JNK Proteins.**

Sarin Chimnaronk^1^ , Jatuporn Sitthiroongruang^1^, Kanokporn Srisucharitpanit^2^, Monrudee Srisaisup^1^, Albert J. Ketterman^1^, Panadda Boonserm^1*^

^1^ Institute of Molecular Biosciences, Mahidol University, Salaya, Phuttamonthon, Nakhon Pathom 73170, Thailand

^2^ Faculty of Allied Health Sciences, Burapha University, Saen Sook, Mueang District, Chonburi 20131, Thailand

E-mails: Sarin Chimnaronk - sarin.chim@mahidol.ac.th; Jatuporn Sitthiroongruang - robben16_chelsea@hotmail.com; Kanokporn Srisucharitpanit - kanokporns@buu.ac.th; Monrudee Srisaisup – catta_w255@hotmail.co.th; Albert J. Ketterman - albert.ket@mahidol.ac.th; Panadda Boonserm* - panadda.boo@mahidol.ac.th

*Corresponding author

*Drosophila* APLIP1 -----------------------------------MADSEFEEFHR-------------- 11

Mammalian JIP1 MAERESGGLGGGAASPPAASPFLGLHIASPPNFRLTHDISLEEFEDEDLSEITDECGISL 60

* ***

Drosophila APLIP1 ------PIFEPHTIAGFGSGAGSKKNNPHAFYSLIPNDDLEDSHSSKSDGDGSDQEDGIG 65

Mammalian JIP1 QCKDTLSLRPPRAGLLSAGGGGAGSRLQAEMLQMDLIDATGDTPGAEDDEEDDDEERAAR 120

* * * * * * * *

*Drosophila* APLIP1 LVDHEPKMRQVEDDELGDGLKVTLSSDGSLDTNDSFNSHRHHPLNHQDAIGG-------- 117

Mammalian JIP1 RPGAGPPKAESGQEPASRGQGQSQGQSQGPGSGDTYRPKRPTTLNLFPQVPRSQDTLNNN 180

* * * * **

*Drosophila* APLIP1 -------------------------------FLGMDTSGLGGNSAPVTIGASTDLLAPNT 146

Mammalian JIP1 SLGKKHSWQDRVSRSSSPLKTGEQTPPHEHICLSDELPPQSGPAPTTDRGTSTDSPCRRS 240

* * * ***

*Drosophila* APLIP1 AATRRR------------------------------------------------------ 152

Mammalian JIP1 TATQMAPPGGPPAAPPGGRGHSHRDRIHYQADVRLEATEEIYLTPVQRPPDAAEPTSAFL 300

**

*Drosophila* APLIP1 ---------------RKLPEIPKNKKSSILHLLGGSNFGSLADEFRNGGG------GGIP 191

Mammalian JIP1 PPTESRMSVSSDPDPAAYPSTAGRPHPSISEEEEGFDCLSSPERAEPPGGGWRGSLGEPP 360

* ** * * ** * *

*Drosophila* APLIP1 PAVRSGQQ-----RSFLSLKCGYLMDEDSSPDSERMQSLG-------------------- 226

Mammalian JIP1 PPPRASLSSDTSALSYDSVKYTLVVDEHAQLELVSLRPCFGDYSDESDSATVYDNCASVS 420

* * * * * **

*Drosophila* APLIP1 -----DVDSGHSTAHSPNDFKSMSPQITSPVSQSPFPPPFGGVPFG-------------- 267

Mammalian JIP1 SPYESAIGEEYEEAPRPQPPACLSEDSTPDEPDVHFSKKFLNVFMSGRSRSSSAESFGLF 480

* * * * * * *

*Drosophila* APLIP1 ----QLEMLEATHRGLHKFVPRHHDEIELEIGDAIYVQKEAEDLWCEGVNLRTGRQGIFP 323

Mammalian JIP1 SCIINGEEQEQTHRAIFRFVPRHEDELELEVDDPLLVELQAEDYWYEAYNMRTGARGVFP 540

* * *** ***** ** *** * * *** * * * *** * **

*Drosophila* APLIP1 SAYAVDLDYNEFDPTVQLVKK----ERYLLGYLGSVETLAHKGTGVVCQAVRKIVGEYGN 379

Mammalian JIP1 AYYAIEVTK-EPEHMAALAKNSDWVDQFRVKFLGSVQVPYHKGNDVLCAAMQKIATTRRL 599

** * * * **** *** * * * **

*Drosophila* APLIP1 SPTG---QTCILEVSDQGLRMVDRSGPNQNKKDKKPCIDYFYSLKNVSFCAFHPRDHRFI 436

Mammalian JIP1 TVHFNPPSSCVLEISVRGVKIGVKADDSQEAKGNK--CSHFFQLKNISFCGYHPKNNKYF 657

* ** * * * * * * *** *** **

*Drosophila* APLIP1 GFITKHPTVQRFACHVFKGSESTRPVAEAVGRAFQRFYQKFIETAYPIEDIYIE 490

Mammalian JIP1 GFITKHPADHRFACHVFVSEDSTKALAESVGRAFQQFYKQFVEYTCPTEDIYLE 711

******* ******* ** ** ****** ** * * * **** *

**Additional file 2**. Pairwise sequence alignment of mammalian JIP1 and *Drosophila melanogaster* APLIP1 [UniProt:Q9UQF2 and UniProt:Q9W0K0, respectively] with amino acid sequence identity of about 26%. A putative docking motif could be mapped at positions 102-112 of APLIP1 when compared to that of the mammalian JIP1 as shown in grey highlight. Conserved amino acids are denoted by asterisks.
